# Perinatal Exposure to Low Levels of the Environmental Antiandrogen Vinclozolin Alters Sex-Differentiated Social Play and Sexual Behaviors in the Rat

**DOI:** 10.1289/ehp.7509

**Published:** 2005-03-16

**Authors:** Nathan K.W. Colbert, Nicole C. Pelletier, Joyce M. Cote, John B. Concannon, Nicole A. Jurdak, Sara B. Minott, Vincent P. Markowski

**Affiliations:** Maine Center for Toxicology and Environmental Health, University of Southern Maine, Portland, Maine, USA

**Keywords:** antiandrogen, penile reflexes, prenatal exposure, rat, social play, vinclozolin

## Abstract

In this study we examined the effects of exposure to the antiandrogenic fungicide vinclozolin (Vz) on the development of two sex-differentiated behaviors that are organized by the perinatal actions of androgens. Pregnant Long-Evans rats were administered a daily oral dose of 0, 1.5, 3, 6, or 12 mg/kg Vz from the 14th day of gestation through postnatal day (PND)3. The social play behavior of juvenile offspring was examined on PND22 and again on PND34 during play sessions with a same-sex littermate. After they reached adulthood, the male offspring were examined with the *ex copula* penile reflex procedure to assess erectile function. Vz did not produce any gross maternal or neonatal toxicity, nor did it reduce the anogenital distance in male pups. We observed no effects of Vz on play behavior on PND22. However, the 12-mg/kg Vz dose significantly increased play behavior in the male offspring on PND34 compared with controls. The most dramatic increases were seen with the nape contact and pounce behavior components of play. The Vz effect was more pronounced in male than in female offspring. As adults, male offspring showed a significant reduction of erections at all dose levels during the *ex copula* penile reflex tests. The 12-mg/kg dose was also associated with an increase in seminal emissions. These effects demonstrate that perinatal Vz disrupts the development of androgen-mediated behavioral functions at exposure levels that do not produce obvious structural changes or weight reductions in androgen-sensitive reproductive organs.

Fungicides are applied to many foods to control plant diseases such as *Sclerotinis sclerotiorum* (white mold) and *Botrytis cinerea* (gray mold) (Papadopoulou-Mourkidou 1991). After application, fungicides have been shown to volatilize and circulate through air and water and on untreated foods, increasing their distribution (Baumeister et al. 2002). Consumers cannot readily reduce their exposure because fungicides are not removed from fresh produce by rinsing with tap water (Krol et al. 2000), and commercial processing increases their concentrations (Will and Kruger 1999). Fungicides are also widely used on golf courses, industrial landscapes, lawn turf, and ornamental plants, where they can enter water supplies in contaminated runoff (Haith and Rossi 2003).

The dicarboximide fungicide vinclozolin (Vz) is used in a number of commercial formulations to treat fruits and vegetables such as lettuce, snap beans, canola, and grapes [U.S. Environmental Protection Agency (EPA) 2003]. Vz belongs to a group of environmental endocrine disruptors known as the anti-androgens. These compounds share a common, clearly defined hormone-receptor–mediated mechanism of action. Vz is biotransformed into at least two active metabolites that bind competitively to the human, monkey, and rat androgen receptor (Kelce and Monosson 1995; Kelce et al. 1994). Exposure to antiandrogens during development could have serious effects on sexual development. They are already recognized as one of the factors responsible for the recent increase of hypospadias, a male reproductive disorder where the urethral opening is on the ventral surface of the penis (Baskin et al. 2001; Davis et al. 1998; Egeland et al. 1994; Jensen et al. 1995; Sharpe and Skakkebaek 1993).

To date, most investigations have focused on the impact of the environmental anti-androgens on the development of androgen-sensitive male reproductive organs. For example, adult male rats have reduced anogenital distances (AGDs), reduced seminal vesicle and ventral prostate weights, and lower epididymal sperm counts after perinatal exposure to Vz (Gray et al. 1994; Hellwig et al. 2000). Vz is not the only fungicide that acts as an environmental antiandrogen. Procymidone and iprodione are fungicides that are structurally similar to Vz, and they produce a nearly identical profile of effects on the reproductive system (Gray et al. 1999b; Ostby et al. 1999). All three fungicides have the same final metabolite. However, the doses of Vz that have been shown to affect the weights of reproductive organs in animal studies are quite high, often 5–10 times the U.S.EPA’s lowest observed adverse effect level (LOAEL). High doses are associated with measures of gross toxicity such as lowered body weights and increased mortality rate due to granulomas and bladder stones (Gray et al. 1994; Hellwig et al. 2000). Thus, although effects such as reductions in rat reproductive organ weights demonstrate that environmental antiandrogens can produce long-term effects after perinatal exposure, they do not necessarily represent the most sensitive end points. Investigations of the functional effects of low-level environmental antiandrogen exposure are needed to complement the high-dose studies and place organ deficits into the larger context of male reproductive health.

One of the questions examined in the present study was whether much lower levels of Vz during the perinatal period affect reproduction via a disruption of male copulatory behavior. An androgen-sensitive neuro-muscular system that is critical for normal male copulatory behavior is the levator ani and bulbocavernosus (BC) skeletal muscles and their motor neuron control centers in the lumbar spinal cord [the spinal nucleus of the BC (SNB)]. In rats, contraction of the levator ani and BC muscles, as well as vascular mechanisms, produces penile erections (Hart and Melese-D’Hospital 1983; Leipheimer and Sachs 1993; Sachs 1982). The sex-specific development of the BC/SNB system is organized during the perinatal period by the non-aromatizable androgen dihydrotestosterone (DHT) (Hart 1979; Thomas et al. 1982). In developing males, the presence of DHT reduces motor neuron death in the SNB and promotes retention of the BC (Mills and Sengelaub 1993). In the adult male, there are two to three times more SNB motor neurons than in females (Sengelaub et al. 1989). However, environmental antiandrogen exposure can disrupt the development of the SNB/BC system. Vz exposure during the perinatal (Wolf et al. 2000, 2004) or peripubertal period (Monosson et al. 1999) significantly reduces the weight of the BC and levator ani muscles in adult males. Other antiandrogens such as procymidone, prochloraz, and linuron also affect the development of the BC muscle (Lambright et al. 2000; Ostby et al. 1999; Vinggaard et al. 2002).

What are the functional implications of an underweight BC muscle that has been affected by Vz exposure? Gray et al. (1994) have shown that adult male rats exposed to perinatal Vz will mount sexually receptive females but are unable to achieve vaginal penetration, suggesting that there is an underlying erectile dysfunction. Other environmental antiandrogens, such as *p*,*p*′-dichlorodiphenyldichloroethylene (*p*,*p*′-DDE), have already been shown to reduce erectile functions in rats (Brien et al. 2000). Female rats can detect subtle behavioral deficits and prefer to copulate with healthy, dominant males (McClintock et al. 1982). Antiandrogens could therefore affect the reproductive success of a wide range of animal species by altering male copulatory behavior. For instance, female guppies prefer males with high rates of sexual display, and Vz exposure has been shown to significantly reduce male guppy courtship display (Baatrup and Junge 2001; Bayley et al. 2002).

Most functional investigations of environmental endocrine disruptors have focused on the effects of perinatal exposure in adult offspring and have ignored the developmental trajectory of the effects of antiandrogen exposure. Juvenile play is a sexually dimorphic behavior that is an important precursor to adult sexual behavior (Pellis et al. 1992) and dominance relationships (Pellis and Pellis 1992). Males typically engage in more bouts of play and perform more behaviors during bouts than females (Thor and Holloway 1983). Even though they are prominent at different times in the life span, juvenile play and copulation are interconnected. During play, rats perform numerous crawl-over behaviors with same-sex partners. There is a shift of interest in male pups in their preferred play partners during the postnatal day (PND)36–40 period, from male to female (Meaney and Stewart 1981). Older, sexually mature but naive males will perform crawl-overs with females until they achieve a mount with a successful vaginal intromission. After the first intromission, mounting behavior increases and crawl-overs decrease. Adult males that do not have the opportunity to experience normal play during development show excessive play components but little normal copulatory behavior in the presence of sexually receptive females (Gerall et al. 1967; Goldfoot 1977; Gruendel and Arnold 1960; Hard and Larsson 1968). Thus, early environmental factors can affect important neonatal or juvenile social interactions, culminating in aberrant behaviors in adulthood (Dunlap et al. 1978).

The neural mechanisms for play are potential targets for environmental antiandrogens because they are organized in part by androgens during the perinatal period. Administration of the androgen receptor antagonist flutamide during the first 10 postnatal days demasculinizes male rat play behavior (Meaney et al. 1983). Other manipulations such as prenatal protein deprivation (Almeida et al. 1996), perinatal genistein exposure (Flynn et al. 2000), prenatal polychlorinated biphenyl (PCB) exposure (Vreugdenhil et al. 2002), or maternal stress during gestation can also demasculinize play behavior (Ward and Stehm 1991). Recently there has been some interest in the effects of perinatal Vz exposure on social play behavior in juvenile subjects. Hotchkiss et al. (2003) exposed neonatal rat pups to 200 mg/kg Vz on PND2 and PND3. This acute, high-dose exposure had long-term consequences for male rats. Juveniles performed significantly fewer chase and pin behaviors during play sessions with a same-sex partner. Female pups were not exposed to Vz in this study, although it is well known that female play can also be altered by neuro-endocrine manipulations (Hines 2003; Nordenstrom et al. 2002; Servin et al. 2003). One study that used a chronic, low-level dietary exposure to Vz found that female rats were more sensitive than males (Flynn et al. 2001).

In the present study, pregnant rats were exposed to low doses of Vz through the last third of gestation and for several days after parturition. Play behavior was examined in juvenile male and female offspring. Erectile function in adult males was assessed using the *ex copula* penile reflex procedure.

## Materials and Methods

### Breeding and exposure.

Long-Evans hooded rats (Harlan, Indianapolis, IN) were allowed to acclimate to the University of Southern Maine Vivarium quarters for 2 weeks before breeding. All rats were fed standard pellet chow (Teklad Global 18% Protein Rodent Diet; Harlan Teklad, Madison, WI) *ad libitum* and were maintained on a 12-hr light/12-hr dark cycle in a barrier facility room with an ambient temperature of 68 ± 2°F and 40–60% humidity.

Groups of three females were housed with stud males, and vaginal smears were examined each morning for the presence of sperm. We regarded a sperm-positive smear as gestation day (GD)0. Pregnant rat dams were placed individually into polycarbonate shoebox cages and assigned to an exposure condition according to a randomized block design. Each block consisted of five groups: 0, 1.5, 3, 6, or 12 mg Vz/kg maternal body weight. Vz (Crescent Chemical Co. Inc., Islandia, NY) was dissolved in corn oil, and the appropriate volume (~ 0.5–1.5 mL) was administered via gavage from GD14 through PND3 to coincide with the period of sexual differentiation in the rat (Miller et al. 1988). Vz was not administered on the day of parturition (PND0). We chose the doses in order to examine a range below the U.S.EPA’s LOAEL of 11.5 mg/kg/day (U.S. EPA 2000a). The adverse developmental event that is associated with the LOAEL is the retention of nipples and areolas in immature male offspring.

We recorded maternal body weights daily during the gestational period. Cages were inspected each morning and afternoon for the presence of litters. Litter size, sex distribution, pup weights, and AGDs were recorded on PND1 and every 4 days thereafter. Using a randomized procedure, litters were culled to eight offspring on PND4, maintaining equivalent sex distributions when possible. After weaning on PND21, offspring were housed with same-sex littermates in plastic cages with filter bonnets. All animal procedures complied with approved institutional animal care protocols and in accordance with National Institutes of Health guidelines (Institute of Laboratory Animal Resources 1996). Animal care and welfare were supervised by a veterinarian and a Registered Laboratory Animal Technologist certified by the American Association of Laboratory Animal Science.

The exposure and rearing procedure yielded a total of 51 viable litters (Table 1). From this cohort, we assigned 11, 11, 8, and 6 pairs of same-sex littermates from the 0-, 3-, 6-, and 12-mg/kg groups, respectively, to the play procedure. Only those litters with at least 2 male offspring and 2 female offspring were assigned to this procedure. The litter was always considered the unit of analysis, and only 1 male and 1 female pair per litter was assigned to the play procedure. For the penile reflex procedure, 11, 7, 11, 10, and 6 male offspring from the 0-, 1.5-, 3-, 6-, and 12-mg/kg groups were assigned, respectively.

### Play behavior.

We randomly selected two male and two female pups from each litter. Within each same-sex pair, one animal was designated as the “target” and the other animal served as partner. Before data collection, target and partner animals were marked with a nontoxic marker for identification; they were then separated from their littermates. Twenty-four hours later, the target animal and their same-sex partner were placed together for 10 min in a glass aquarium (12 in. wide ×24 in. long × 12 in. high) with clean cage bedding. We filmed their interactions under dim red light with a Canon XL1s digital video camera (Canon, Inc., Lake Success, NY) interfaced to an iMac computer running iMovie software (Apple Computer, Inc., Cupertino, CA). All testing was conducted during the middle of the dark phase of the light/dark cycle. No other animals were present in the room during filming.

We examined play behavior on PND22 and again on PND34 in the same animals. The assessment ages were chosen in order to examine play immediately after weaning on PND21 and immediately before the decline in same-sex play in male rats, which begins during PND36–40 (Meaney and Stewart 1981).

A pair of trained observers later viewed the films using QuickTime software. (Apple Computer Inc.) Observers tabulated the frequency and distribution of the following five behaviors: nape attack (the snout of the target animal makes contact with the nape area of the partner animal; this behavior occurs frequently and often initiates a bout of play behavior); pounce (the target animal lunges forward with its forepaws extended and makes contact with the partner animal); pin (the target animal is positioned on top of the partner animal with its forepaws placed on the partner; the partner animal lies on its back, fully exposing its ventral surface to the target animal); wrestle (the target and partner animal roll and tumble with each other); and mount (a component of the adult male copulatory pattern where the target animal approaches the partner animal from the rear, clasps its flanks, and mounts).

### Penile reflex.

In rats, reflexive penile erections and movements can be observed if the penile sheath is retracted with light pressure directed at the base of the penis (Hart and Melese-D’Hospital 1983; Sachs and Garinello 1978). Penile reflexes in the rat consist of erections (tumescence followed by detumescence), cupping (the end of the erect glans penis flares out), and flipping (rapid dorso-flexion of the erect penis). Erections serve to extend the penis beyond the penile sheath, a function that is necessary to achieve vaginal penetration during copulation. Penile flipping serves to stretch the vaginal wall and cupping serves to collect coagulating semen and seal the seminal plug against the cervix.

We conducted all erection tests during the middle of the dark phase. Tests lasted for 20 min after the first response or for 15 min in the absence of responses. During each test, animals were restrained in a supine position with their head and upper torso positioned in a darkened, ventilated tube (8.5 × 5.5 × 20 cm) fastened to a plastic base. The darkened tube is anxiolytic, and rats rapidly habituate to brief periods of restraint. The penile sheath was retracted and held in place (Hart and Melese-D’Hospital 1983; Sachs and Garinello 1978). Typically, clusters of penile erections and dorsoflexions (movements or “flips”) begin spontaneously within 5–10 min after sheath retraction.

Trained observers recorded the frequency and time distribution of three gradations of erections: E1, reddening and distension of glans; E2, tumescence of the base and tip of glans; and E3, intense erection accompanied by cupping of the tip of glans (Eaton et al. 1991; Hull et al. 1991; Warner et al. 1991). Penile movements, seminal emissions, latency to the first reflex, and the number of response clusters were also determined. We defined a response cluster as any display of responses separated by ≥ 15 sec. Seminal emissions were defined as the expulsion of seminal fluid followed by a coagulating plug.

### Statistical methods.

For the play procedure, the five behaviors were summed and analyzed as total play behaviors per session with repeated-measures analysis of variance (ANOVA). The individual behaviors were also analyzed separately. The litter always served as the statistical unit of analysis, with the exposure level as a between-litter factor and sex and PND as within-litter factors. In cases where there was a significant main effect or interaction involving the exposure factor, Duncan’s probe tests were used to make pairwise comparisons. We considered *p* < 0.05 statistically significant.

For the penile reflex procedure, we tested each animal on 2 consecutive days, and the data from the two sessions were averaged before analysis. If an animal was inactive during one of the sessions, that session was dropped. If an animal was inactive during both sessions, that animal was dropped from the analysis. Only 2 of 47 animals were inactive during both sessions. The averaged behavior was analyzed with one-way ANOVA according to exposure level.

Because the penile erection data showed a clear dose–response relationship with evidence that even the lowest dose of Vz disrupted the behavior, we further examined the data with Benchmark Dose Modeling Software (BMDS; version 1.3.2; U.S.EPA National Center for Environmental Assessment, Washington, DC). BMDS is a useful alternative to the no observed adverse effect level (NOAEL) approach because it uses the entire dose–response relationship and does not involve extrapolations far below experimental observations. We used the BMDS continuous model to calculate benchmark doses that represented the model-estimated control mean, minus proportional deviations equivalent to a 10% (ED_10_) or 1% (ED_01_) decrement in behavior. BMDS also provided a 95% lower bound that can be divided by a standard uncertainty factor to calculate a reference dose or generate a margin of exposure.

## Results

### Maternal and postpartum data.

We found no evidence of gross maternal or neonatal toxicity (Table 1), nor were there any exposure-related changes in maternal body weight, pup body weight, or AGD (males, Table 2; females, Table 3). The percentage of male pups that possessed at least one visible areola on PND12 (control, 18%; 1.5 mg/kg, 50%; 3 mg/kg, 44%; 6 mg/kg, 45%; 12 mg/kg, 33%) was not significant (*p* = 0.367).

### Play behavior.

**Figure 1:**
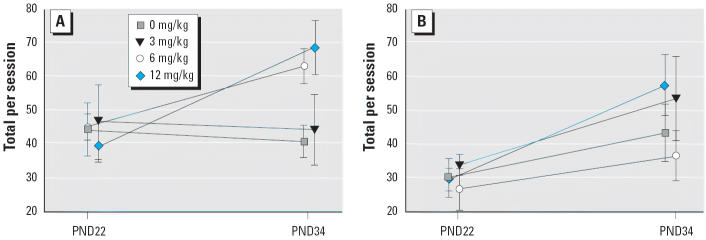
Mean ± SEM total number of play behaviors per session on PND22 and PND34 for male offspring (*A*) and their female littermates (*B*). Males exposed to 6 mg/kg or 12 mg/kg Vz performed significantly more behaviors on PND34 than did same-sex controls; females were not significantly different on either day.

For this procedure, the primary dependent variable was the total number of play behaviors per session. Although we found no significant main effects of the exposure, sex, or PND factors on total play behaviors, there was a significant exposure × PND interaction [*F*(1,21) = 7.72, *p* = 0.01]. We also examined the total number of behaviors separately for PND22 and PND34. There was a significant effect of sex on PND22 [males > females; *F*(1,21) = 9.91, *p* < 0.01] and a significant effect of exposure on PND34 [*F*(1,37) = 16.38, *p* < 0.001]. Probe tests revealed that the male 12-mg/kg and 6-mg/kg Vz groups produced significantly more play behaviors than the did controls on PND34 (Figure 1A). There were no differences between the female exposure groups (Figure 1B).

**Figure 2:**
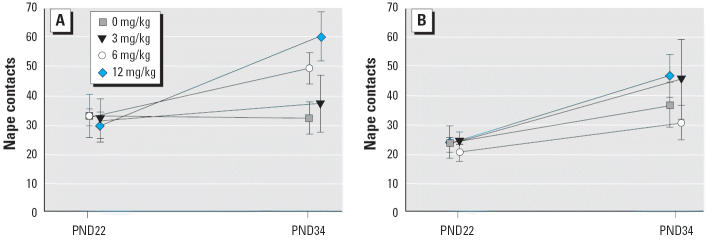
Mean ± SEM number of nape contacts per session on PND22 and PND34 for male offspring (*A*) and their female littermates (*B*). The male 12-mg/kg Vz group performed significantly more nape contacts on PND34 than did controls or the 3-mg/kg group.

**Figure 3:**
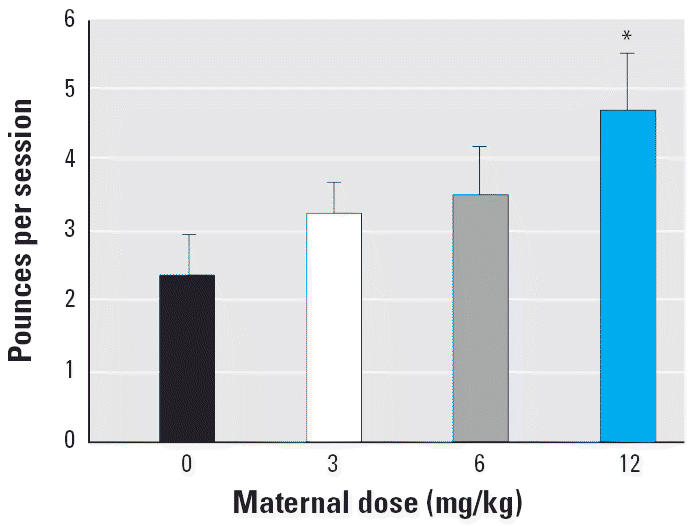
Mean ± SEM number of pounces per session. Data have been averaged across sex and postnatal age groups. The 12-mg/kg Vz group produced pounced significantly more than did the control group.
*p < 0.05.

Nape contact and pounce variables made the greatest contribution to the significant exposure-related effects on total play behaviors. For nape contacts, there was a significant exposure × PND interaction [*F* (1,21) = 5.51, *p* = 0.03]. We also examined the number of nape contacts separately for PND22 and PND34. As with the total play behavior variable, there was a significant main effect of sex on PND22 [males > females; *F*(1,21) = 11.13, *p* < 0.01] and a significant main effect of exposure on PND34 [*F*(1,37) = 16.09, *p* < 0.001]. Probe tests indicated that the male 12-mg/kg Vz group produced significantly more nape contacts than did the 0- and 3-mg/kg groups on PND34 (Figure 2). For the pounce variable, there was a significant main effect of exposure [*F*(1,21) = 6.44, *p* = 0.02]. Data were averaged across sex and age, and probe tests indicated that the 12-mg/kg group pounced more than did controls (Figure 3). There were no exposure-related differences for pin, wrestle, or mount behaviors.

### Penile reflex.

**Figure 4:**
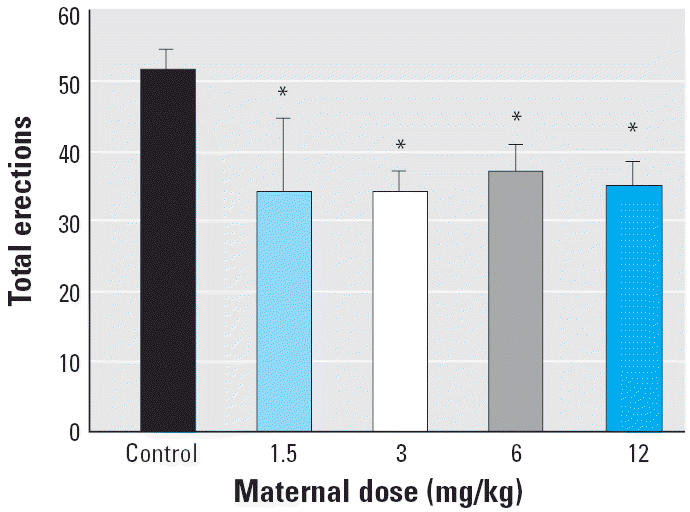
Mean ± SEM total number of erections during the *ex copula* penile reflex procedure. All four of the Vz exposure groups produced significantly fewer erections than did the control group.
*p < 0.01.

**Figure 5:**
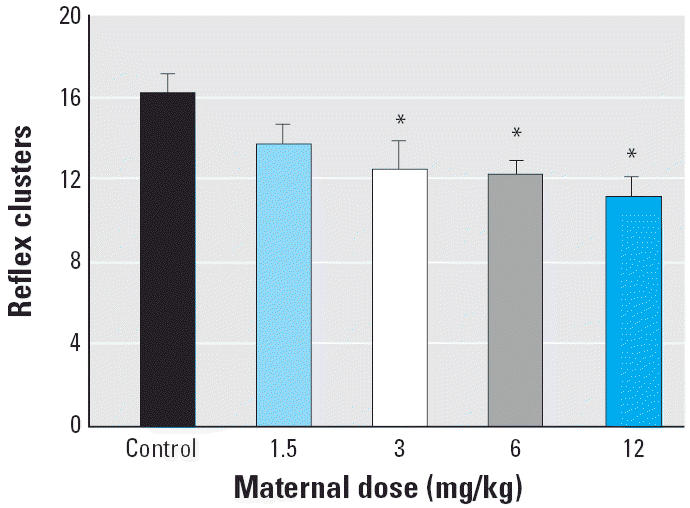
Mean ± SEM total number of reflex clusters during the *ex copula* penile reflex procedure. A cluster of reflexes is defined as a rapid sequence, where each behavior occurs within 15 sec of the previous behavior. The 3-, 6-, and 12-mg/kg Vz groups produced significantly fewer clusters of reflexes than did the control group.
*p < 0.01.

**Figure 6:**
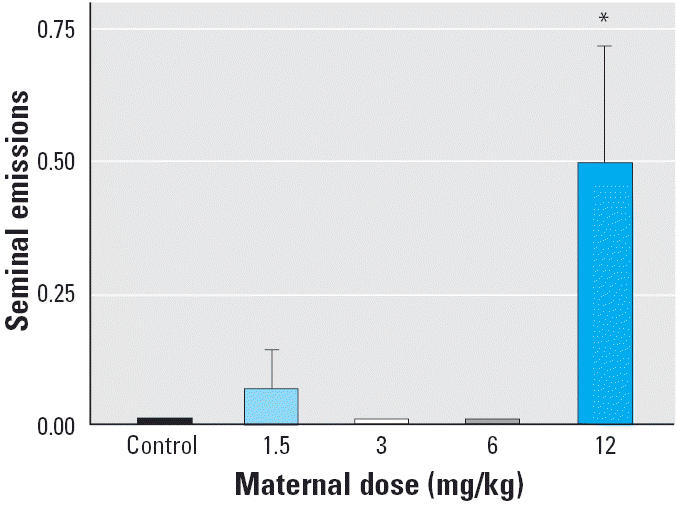
Mean ± SEM total number of seminal emissions during the *ex copula* penile reflex procedure. The 12-mg/kg Vz group produced significantly more emissions than did the control group.
*p = 0.02.

We found a significant exposure-related decline in total erections per session [*F*(4,40) = 4.62, *p* < 0.01; Figure 4] as each of the Vz groups produced significantly fewer erections than controls. The decline in total erections was due primarily to a dose-related decline of E1 or low-intensity erections [*F*(4,40) = 10.07, *p* < 0.01] as well as the number of reflex clusters per session [*F*(4,40) = 3.23, *p* = 0.02; Figure 5]. The latency to the first penile reflex and the frequency of E2 and E3 responses were not significantly different. Surprisingly, there was a significant increase in seminal emissions [*F*(4,40) = 7.37, *p* < 0.01; Figure 6] as the 12-mg/kg group expelled more often than did any of the other groups. This effect was unanticipated because rats do not usually emit seminal fluid during the *ex copula* procedure.

### Benchmark dose modeling.

**Figure 7:**
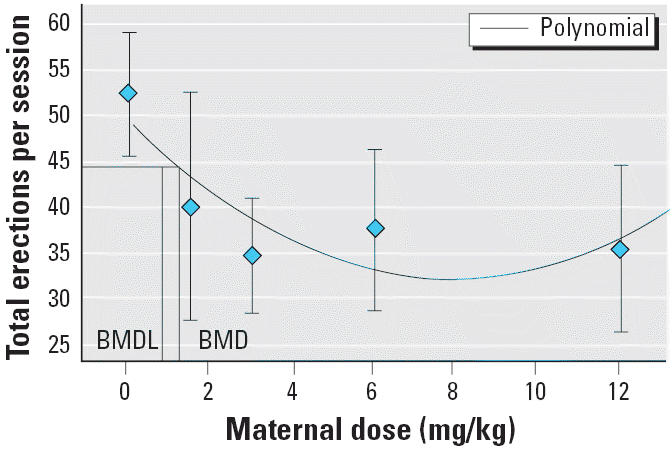
Polynomial model for ED_10_ benchmark dose (BMD) value and 95% lower confidence level (BMDL) for the total erections per session variable.

**Figure 8:**
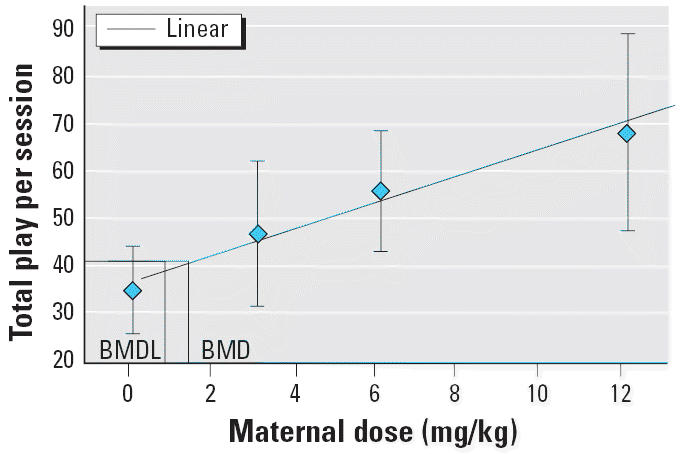
Linear model for ED_10_ benchmark dose (BMD) value and 95% lower confidence level (BMDL) for total play behavior in the male offspring on PND34.

We performed benchmark dose calculations on the total erections per session and the total play behavior in the male offspring on PND34. These two variables were selected because they are the best overall measures of erectile function and play, and consequently, they generalize more readily to humans. A polynomial model provided the best description of the erection data. The corresponding ED_10_ benchmark for erections was 1.23 mg/kg with a 95% lower bound of 0.84 mg/kg (Figure 7). The ED_01_ benchmark was 0.11 mg/kg with a lower bound of 0.08 mg/kg. The linear model provided the best description of the play data. The ED_10_ associated with total play in the male offspring on PND34 was 1.33 mg/kg with a lower bound of 0.77 mg/kg (Figure 8). The ED_01_ for total play was 0.13 mg/kg with a lower bound of 0.08 mg/kg.

## Discussion

The results of this study clearly demonstrate that social and reproductive behaviors in the rat are disrupted by exposure to low doses of Vz during the perinatal period. Maternal doses of 12 mg/kg, administered from GD14 through PND3, were associated with a significant increase in social play behavior in PND34 offspring. We observed no Vz-mediated differences on PND22, indicating that the effect emerged as offspring matured. The increased play on PND34 was more pronounced in males than in females. In adulthood, male offspring produced significantly fewer penile erections, an effect that was even more sensitive than play behavior because a decrease was noted after maternal doses as low as 1.5 mg/kg.

Although other researchers have reported that high doses of Vz (200 mg/kg) administered to rat pups on PND2 and PND3 reduced play behavior (Hotchkiss et al. 2003), this study is the first to describe play behavior effects near the LOAEL of 11.5 mg/kg/day (U.S. EPA 2000a). Although we did not observe nipple and areola retention in immature male offspring, visual inspection of the data suggests that there was a dose-related trend.

The play behavior procedure used in the present study was more sensitive to low-dose effects than those used in previous investigations, possibly because of methodologic differences. In the present study we examined nape contact, pounce, pin, and wrestle, as well as mount behaviors, whereas previous studies examined only pin (Flynn et al. 2001) or pin and chase behaviors (Hotchkiss et al. 2003). Nape contact, a behavior that often initiates a play bout, was greatly affected by perinatal Vz, and this component was not examined in previous studies. Although we hypothesized that perinatal Vz would demasculinize male offspring and lead to a reduction of play behavior, we actually observed a dose-related increase in play. Exposure to other developmental toxicants such as prenatal morphine (Hol et al. 1996; Niesink et al. 1999), mycotoxins (Ferguson et al. 1997), or phytoestrogens (Flynn et al. 2000) has been associated with increased play, and, as mentioned above, social hyperactivity in juvenile rats is linked to aberrant sexual behavior in adults (Gerall et al. 1967).

In the present study we also examined play behavior at two different time points, an approach that detected the apparently transient or age-specific effect of perinatal Vz. In rats, the ontogeny of play is characterized by an inverted U-shaped function that peaks between PND32 and PND40 (Panskeep 1980; Spear and Brake 1983; Ward and Stehm 1991). Male behaviors peak earlier, during PND26–35, with female behaviors peaking during PND36–40 (Meaney and Stewart 1981). The increased play observed in the PND34 exposed males could be interpreted as a developmental delay. Peripubertal exposure to higher doses of Vz has been shown to delay the age of preputial separation, which is a milestone of puberty in the male rat (Monosson et al. 1999). Typically, as male rats age, they show an increasing preference for female versus male partners, a shift that was not observed in the juvenile Vz males. The behavior of the exposed males actually resembled the female offspring, who performed more play on PND34 than on PND22. Future studies should examine additional time points to better characterize the age-dependent nature of the effects of Vz on play. Alternatively, the increased play in the exposed offspring could be due to greater sensitivity to social isolation. Because social deprivation is often viewed as a means of increasing play motivation, this hypothesis could be explored in future studies that compare play after different periods of deprivation. In normal juvenile rats, play solicitation increases after longer periods of deprivation (Thor and Holloway 1983).

Finally, it might be the case that we found significant effects at lower doses in the present study because the offspring were exposed during gestation and the neonatal period via maternal dosing with the gavage procedure. Although many play behavior studies have focused on the role of androgens during the neonatal period, perhaps because of the ease of working with newborn versus fetal rats, the available evidence suggests that the critical period for the differentiation of play begins late in gestation and continues through PND10 (Meaney et al. 1983; Ward and Stehm 1991). Data on the effects of prenatal morphine suggest that the onset of the critical period for play is GD16 (Niesink et al. 1999).

A survey of the developmental toxicology literature indicates that the reduction of erections measured in the present study is one of the most sensitive outcomes observed to date in a perinatal Vz study. Earlier work found that reduced AGD in male neonates and nipple retention occurred after exposure to maternal doses as low as 3.125 mg/kg, whereas at least 50 mg/kg was required to affect ventral prostate weight and increase the incidence of hypospadias (Gray et al. 1999a). Although behavior analysis was not an objective in these earlier investigations, during copulation, exposed males mounted but were unable to achieve vaginal penetration (Gray et al. 1994). In adult male rats, a number of manipulations can produce similar effects, including castration (Leipheimer and Sachs 1993), lesions of the medial preoptic area (Everitt 1990), or microinjection of dopamine antagonist drugs (Pfaus and Phillips 1991). In developing males, prenatal exposure to antiestrogens (Matuszczyk and Larsson 1995) also appears to impair copulatory performance without disturbing sexual motivation. All of these procedures produce structural or functional changes in the erectile system (Hull et al. 1992; Monaghan et al. 1993; Warner et al. 1991).

The Vz-exposed males showed a selective reduction of low-intensity (E1) erections. In this regard, the exposed offspring resemble males castrated as adults, which also show an early reduction of E1 erections (Leipheimer and Sachs 1993). The behavior of the exposed offspring is also reminiscent of male rats that have been administered serotonin receptor agonists (Mas et al. 1985) or agents that block the synthesis of nitric oxide (Hull et al. 1994). Both of these treatments reduce erections and increase seminal emissions. Perhaps the most parsimonious explanation of the differential regulation of penile erections versus seminal emissions has been offered by Hull and others. In a series of drug microinjection studies, this group has demonstrated that pharmacologic stimulation of dopamine D2 receptors in the medial preoptic area decreases the frequency of erections while increasing seminal emissions (Bazzett et al. 1991). Stimulation of D2 receptors in the paraventricular nucleus also facilitates seminal emission (Eaton et al. 1991; Pehek et al. 1989). On the other hand, stimulation of D1 receptors in the medial preoptic area has the opposite effect and occurs at much lower doses (Hull et al. 1992). Because the functional integrity of dopamine systems in this part of the brain is maintained by circulating testosterone (Du and Hull 1999), an environmental antiandrogen such as Vz might disrupt the development of these complex interactions.

As mentioned above, animal studies indicate that fetal males are far more sensitive to environmental antiandrogens than adults. Results from maternal stress studies shed some light on the likely developmental mechanisms affected by environmental antiandrogens. Maternal stress during the last week of pregnancy lowers the surge of plasma testosterone that is normally present in male rat fetuses during GD18 and GD19 (Ward and Weisz 1984). Attenuation of the GD18–19 surge is associated with impaired sexual behavior in adulthood (Dunlap et al. 1978; Ward and Reed 1985). This testosterone surge also exerts an organizational effect on the muscle and spinal cord mechanisms that control penile erections in adulthood (Grisham et al. 1991). Perinatal androgens serve to rescue SNB motor neurons from programmed death (Sengelaub et al. 1989), a process that could be blocked by an antiandrogen like Vz. In the present study, animals were exposed to Vz from GD14 through PND3 in order to compare our results with previous perinatal Vz investigations. However, because differentiation of spinal cord motor neurons continues until PND10 (Mills and Sengelaub 1993) and the weight of the adult BC muscle is the most sensitive to Vz exposure during the GD16–17 period (Wolf et al. 2000), it is likely that the toxic window for Vz on erectile function spans the GD16–PND10 period. Thus, it appears that the critical periods for masculinization of erectile function and play behavior in the male rat are the same. As of yet, no one has examined the effects of environmental antiandrogen exposure during this entire perinatal sensitive period. It may be the case that social play and erectile functions are responsive to even lower doses of Vz, if an exposure were to span the GD16–PND10 period.

No previous studies explicitly link Vz to human erectile dysfunction. However, Vz and other antiandrogenic fungicides are used in agriculture, they may be responsible for the recently noted link between pesticide exposure and erectile dysfunction in otherwise healthy men. Specifically, pesticide-exposed men had a significantly higher incidence of complete impotence, showing little to no change from baseline flaccidity on measures of penile rigidity, tumescence, frequency, and duration (Oliva et al. 2002). Occupational exposure to stilbene has also been associated with an increase in self-reported impotence and decreased libido (Quinn et al. 1990; Whelan et al. 1996). Stilbene is a component of textile finishing agents and detergents, and it is structurally similar to the synthetic estrogen diethylstilbestrol. Both of these clinical studies examined the effects of exposure during adulthood. The long-term effects in men after perinatal exposure are unknown.

It is estimated that children 1–6 years of age are exposed to 0.167 mg Vz/kg body weight/day (U.S. EPA 2000b). Given this chronic exposure estimate, a 2-year-old boy who weighs 13 kg [Centers for Disease Control and Prevention (CDC) 2000] would consume an average of 2.17 mg Vz/day, whereas a 6-year-old with a body weight of 21 kg would consume an average of 3.51 mg Vz/day. Both of these estimated daily intakes exceed the ED_10_ benchmark doses associated with altered juvenile play behavior and erectile dysfunction in our animal model. Typically, the U.S. EPA would divide the 95% lower bound by 100 to calculate a reference dose. If this practice were applied to the juvenile play behavior or erectile data, the average daily intake of Vz would exceed the reference doses based on these data by more than two orders of magnitude. It should also be pointed out that humans are exposed to multiple compounds on a chronic basis, whereas this study examined only Vz, which was administered during a limited period of development. The cumulative effects of chronic exposure to multiple compounds and their metabolites are unknown. Lastly, our benchmark doses should be interpreted as conservative estimates because they are based on maternal doses. The actual amount of Vz and/or metabolite that entered our fetal or neonatal subjects is unknown, although the level was certainly lower than the applied maternal dose.

In conclusion, the results of this study demonstrate that the effects of perinatal exposure to an environmental endocrine disruptor can be observed throughout the life span, provided that age-appropriate, sex-specific end points are examined. Low doses of Vz administered during the GD14–PND3 period significantly increased social play behavior in juvenile male rat offspring. These results are interesting in light of recent findings in humans that higher prenatal levels of PCBs have been associated with less masculinized play behavior in Dutch schoolboys and more masculinized play in schoolgirls (Vreugdenhil et al. 2002). Even lower doses of Vz reduced the erectile response in adult male offspring. Because men who work with agricultural chemicals are more likely to experience erectile dysfunction (Oliva et al. 2002), it is quite possible that some of the relevant agrochemicals are antiandrogenic fungicides.

## Correction

The AGD data for control females were incorrect in Table 3 of the original manuscript published online, but they have been corrected here.

## Figures and Tables

**Table 1:** Mean values for parturition end points for control and Vz-exposed rats.

				Pups per litter		
Exposure group	Sperm-positive females assigned to group*^a^*	Sperm-positive females that delivered a litter	Gestation length (days)	Male	Female	Postnatal mortality
Control	14	12	22.1	6.3	6.3	1
Vz						
1.5 mg/kg	8	7	22.0	4.2	5.7	3
3 mg/kg	16	13	21.9	6.1	6.3	3
6 mg/kg	15	13	22.2	4.7	5.2	2
12 mg/kg	7	7	22.3	6.1	7.1	7*^b^*

a The number of sperm-positive females differs because several females did not copulate or the sperm plug was not detected.

b Five pups were lost from one litter and two from a second litter.

**Table 2:** Mean ± SEM body weight (g) and AGD (mm) for male pups.

No. of litters	Exposure group	End point	PND1	PND4	PND8	PND12	PND16	PND20
12	Control	Body weight	6.9 ± 0.25	10.4 ± 0.52	18.4 ± 0.96	28.3 ± 1.08	36.5 ± 1.34	48.4 ± 1.98
		AGD	3.6 ± 0.39	4.7 ± 0.19	6.7 ± 0.18	9.1 ± 0.29	12.0 ± 0.23	16.5 ± 0.46
7	1.5 mg/kg	Body weight	6.6 ± 0.47	10.4 ± 0.75	16.6 ± 1.6	25.5 ± 1.83	33.3 ± 2.33	43.6 ± 3.28
		AGD	3.4 ± 0.15	4.4 ± 0.17	6.1 ± 0.52	8.5 ± 0.57	12.4 ± 0.87	15.8 ± 1.25
13	3 mg/kg	Body weight	6.7 ± 0.17	10.0 ± 0.25	17.7 ± 0.61	26.9 ± 0.53	35.2 ± 0.67	45.9 ± 0.88
		AGD	3.7 ± 1.0	4.5 ± 0.14	6.5 ± 0.14	9.0 ± 0.25	12.4 ± 0.23	16.3 ± 0.48
13	6 mg/kg	Body weight	7.4 ± 0.27	11.0 ± 0.54	19.2 ± 0.79	28.4 ± 0.74	37.7 ± 1.18	47.5 ± 1.27
		AGD	3.5 ± 0.11	4.8 ± 0.16	6.8 ± 0.20	9.0 ± 0.24	12.0 ± 0.30	16.0 ± 0.47
7	12 mg/kg	Body weight	6.9 ± 0.35	9.6 ± 0.57	19.2 ± 1.01	27.8 ± 1.20	36.7 ± 1.72	47.5 ± 2.79
		AGD	3.7 ± 0.10	4.7 ± 0.11	6.7 ± 0.11	8.7 ± 0.30	12.7 ± 0.54	15.4 ± 0.41

**Table 3:** Mean ± SEM body weight (g) and AGD (mm) for female pups.

No. if litters	Exposure group	End point	PND1	PND4	PND8	PND12	PND16	PND20
12	Control	Body weight	6.6 ± 0.24	9.8 ± 0.51	17.7 ± 0.96	27.3 ± 1.18	35.1 ± 1.37	46.2 ± 1.89
		AGD	2.2 ± 0.04	2.2 ± 0.04	2.7 ± 0.10	4.3 ± 0.16	6.5 ± 0.23	8.5 ± 0.21
7	1.5 mg/kg	Body weight	6.7 ± 0.31	10.6 ± 0.67	17.9 ± 0.69	27.2 ± 0.52	35.2 ± 0.43	45.8 ± 1.25
		AGD	2.1 ± 0.09	2.1 ± 0.09	2.6 ± 0.03	4.2 ± 0.12	6.3 ± 0.22	8.9 ± 0.16
13	3 mg/kg	Body weight	6.4 ± 0.17	9.6 ± 0.26	17.4 ± 0.66	26.3 ± 0.66	34.2 ± 0.79	44.3 ± 1.06
		AGD	2.2 ± 0.04	2.2 ± 0.04	2.7 ± 0.09	4.1 ± 0.14	6.3 ± 0.21	9.0 ± 0.25
13	6 mg/kg	Body weight	6.8 ± 0.21	10.3 ± 0.45	18.0 ± 0.77	26.9 ± 0.57	34.4 ± 0.76	44.9 ± 1.02
		AGD	2.2 ± 0.12	2.2 ± 0.12	2.8 ± 0.07	4.4 ± 0.13	6.5 ± 0.15	8.8 ± 0.19
7	12 mg/kg	Body weight	6.3 ± 0.41	9.0 ± 0.69	17.1 ± 1.22	25.2 ± 1.56	33.8 ± 2.21	43.8 ± 3.31
		AGD	2.2 ± 0.09	2.2 ± 0.09	2.9 ± 0.13	4.2 ± 0.13	6.2 ± 0.18	9.1 ± 0.34
