# Erratum

**Published:** 2006-06

In the article by Colbert et al. [ Environ Health Perspect 113:700–707 (2005)], the authors discovered an error in the units of exposure estimates in the tenth paragraph of the “Discussion.” These estimates are expressed in milligrams but should be expressed in micrograms. The corrected sentences are as follows:

It is estimated that children 1–6 years of age are exposed to 0.167 μg Vz/kg body weight/day …. Given this chronic exposure estimate, a 2-year-old boy who weighs 13 kg … would consume an average of 2.17 μg Vz/day, whereas a 6-year-old with a body weight of 21 kg would consume an average of 3.51 μg Vz/day.

